# Lower Intensified Target LDL-c Level of Statin Therapy Results in a Higher Risk of Incident Diabetes: A Meta-Analysis

**DOI:** 10.1371/journal.pone.0104922

**Published:** 2014-08-14

**Authors:** Rongrong Cai, Yang Yuan, Yi Zhou, Wenqing Xia, Pin Wang, Haixia Sun, Yue Yang, Rong Huang, Shaohua Wang

**Affiliations:** Department of Endocrinology, The Affiliated ZhongDa Hospital of Southeast University, Nanjing, China; Washington Hospital Center, United States of America

## Abstract

**Background:**

A recent meta-analysis has reported that intensive-dose statin drug increases the risk of incident diabetes. However, doubling of the statin dose generates only a further 6% decrease in low-density lipoprotein cholesterol (LDL-c) on average. This study aimed to determine whether statin therapy with lower intensive-target LDL-c level contributes to higher risk of new-onset diabetes.

**Methods:**

Medline, Embase, and the Cochrane Central Register of Controlled Trials were searched for randomized controlled endpoint trials of statins conducted from 1966 to 2012. We included trials with more than 1000 participants who were followed up for at least 2 years. The included trials were stratified by the target LDL-c level. *I* ^2^ statistic was used to measure heterogeneity between trials. We further calculated risk estimates with random-effect meta-analysis. Meta-regression was used to identify the potential risk factors of statin-induced diabetes.

**Results:**

Fourteen trials with a total of 95 102 non-diabetic participants were included. The risks elevated by 33% [odds ratio (OR) = 1.33; 95% confidence interval (CI) 1.14–1.56; *I* ^2^ = 7.7%] and 16% (OR = 1.16; 95% CI 1.06–1.28; *I* ^2^ = 0.0%) when the intensified target LDL-c levels were ≤1.8 mmol/L and 1.8–2.59 mmol/L, respectively. The risk of incident diabetes did not increase when the target LDL-c level was ≥2.59 mmol/L. Apart from age, female, and baseline level of total cholesterol, meta-regression analysis showed that the target and baseline levels of LDL-c and relative LDL-c reduction were predictors of statin-induced diabetes.

**Conclusion:**

A lower intensified target LDL-c level of statin therapy resulted in a higher risk of incident diabetes.

## Introduction

Statin drugs are widely used in the evidence-based lowering of cardiovascular disease (CVD) risk. In a meta-analysis by the Cholesterol Treatment Trialists’ Collaborators of 14 randomized trials of statins involving more than 90 000 participants, statin therapy can safely reduce the 5 year incidence of major vascular events by approximately 20% with per 1.0 mmol/L low-density lipoprotein cholesterol (LDL-c) reduction [1]. Further study concluded that additional lowering of LDL-c to approximately 1 mmol/L to 2 mmol/L with more intensive therapy further reduced the incidence of major vascular events [2]. According to the European Guidelines on CVD prevention in clinical practice (version 2012), the LDL-c goal of <2.6 mmol/L (100 mg/dL) is recommended in high-risk CVD patients. For very high-risk patients, the recommended LDL-c target is <1.8 mmol/L (70 mg/dL) or ≥50% LDL-c reduction when the target LDL-c level cannot be reached [3].

A recent meta-analysis of 13 trials showed that statin therapy is associated with an increased risk of developing type 2 diabetes mellitus (T2DM) [4]. Another meta-analysis combined five trials reported that an excess incidence of new-onset T2DM occurred in patients treated with intensive-dose statin [5]. The intensive-dose statin therapy mentioned above were per day simvastatin 80 mg, atorvastatin 80 mg, pravastatin 80 mg, lovastatin 80 mg, or rosuvastatin 20–40 mg [6]. However, doubling of statin dose generates on average only a further 6% decrease in LDL-c [7]. The guideline requirement for target LDL-c level is difficult to achieve even with high-dose statin drugs. Thus, intensive-dose statin therapy is not the same as intensive-target LDL-c level of statin therapy. Our recent study of eight randomized statin trials investigated the effects of intensive LDL-c lowering with statin drugs (LDL-c level lower than 2.59 mmol/L or relative LDL-c reduction at least 30% of baseline [8]) on new-onset diabetes. The results showed that intensive LDL-c lowering with statin use lead to a 19% increased risk of incident diabetes. Given that an increased trend of incident diabetes appears when LDL-c is lower, we speculate whether the lower target LDL-c achieved contributes to higher risk of incident diabetes. According to the guideline requirement for people at risk of different CVDs [3], a subgroup meta-analysis was conducted in patients whose target LDL-c levels were ≤1.8 mmol/L, within 1.8 mmol/L to 2.59 mmol/L, and ≥2.59 mmol/L. Thus, informed choices were possibly provided by physicians when statin therapy was applied in different kinds of patients. Meanwhile, meta-regression analyses pointed out that statin treatment increases the incidence of new-onset T2DM among old patients, and change in LDL-c concentration is not a risk factor [4]. A study consisted of three large randomized trials of atorvastatin discovered that baseline fasting glucose level, higher triglycerides, higher body mass index (BMI), and hypertension are important predictors of new-onset diabetes [9]. Whether the dose or the target LDL-c level of statin use is associated with diabetes incidence remains unknown.

This study aims to investigate the relationship between target LDL-c level of statin use and new-onset T2DM and identify the potential predictors of statin-induced diabetes. Studies involving 1000 or more patients with a minimum mean follow-up of 2 years and comparison between statin therapy and placebo or standard-care controlled endpoint trials were included.

## Materials and Methods

### Search strategy and selection criteria

The terms statin, hydroxymethylglutaryl coenzyme A reductase inhibitor and names of individual statins (fluvastatin/mevastatin/compactin/pravastatin/simvastatin/lovastatin/Pitavastatin/rosuvastatin/cerivastatin/atorvastatin) combined with diabetes and diabetes mellitus as title words or keywords were searched in Medline (from 1966 to October 2012), Embase (from 1974 to October 2012), and the Cochrane Central Register of Controlled Trials for large-scale trials. These trials compared statin therapy with placebo group or standard care-controlled group.

The search was in strict accordance with Randomized Controlled Trials performed in humans. Two independent reviewers accessed the reports with the following inclusion criteria: trials performed in 1000 or more individuals with a minimum mean follow-up of 2 years. Trials comparing different kinds of lipid-lowering agents or different doses of the same statin or performed in diabetic participants were excluded. The search commenced on October 2012, and 2841 reports were identified. Any discrepancies were settled by discussion with a third reviewer. The improved Jadad score [10]–[12] was used to evaluate the quality of studies included in the meta-analysis.

### Data sources

Of the qualified 23 trials, eight [13]–[20] were available to the authors and six trials [21]–[26] had published data of incident diabetes. For the eight available trials, we referred to another two meta-analyses published in Lancet [4] and JACC [9] for incident diabetes. We inquired the investigators of the other nine trials on the unpublished data for incident diabetes, but no reply was received. A total of 14 trials were included in this study. We also contacted the investigators of some unpublished characteristics of participants (i.e., baseline BMI in LIPID trial [26], mean blood pressure (BP) in LIPID [26] and GISSI PREVENZIONE [20] trials, relative high-density lipoprotein cholesterol (HDL-c) reduction in HPS [22], and GISSI-HF [15] trials). However, we received either rejection or no reply.

The data from the included trials were collected as follows: characteristics of trials (sample size, follow-up), clinical characteristics of the patients (baseline age, gender, current smoker, BMI, BP, HDL-c, LDL-c, triglyceride, and total cholesterol), therapeutic intervention (type and dose of statin), change of serum lipid (endpoint LDL-c level and relative reduction of LDL-c, HDL-c, triglyceride, total cholesterol), other drugs used (aspirin, beta-blocker, and ACE inhibitor), and incident diabetes (including diabetes diagnostic criteria) to identify the risk factors of diabetes (Table 1, Table 2, Table 3, Table 4). A second reviewer checked the extracted data for accuracy.

**Table 1:** Characters for non-diabetic participants in 14 trials that reported incident diabetes.

	Non-DMpatients	Follow-up year	Diagnosis ofincident diabetes	Intervention	NewDMcase	Numbeassignedstatin	Numberin controlgroup	New DMassignedstatin	New DMin controlgroup
JUPITER	17802/17802	1.9‡	Physician reported(medication, positive OGTT,raised random glucosewith symptoms, two fastingglucose values ≥7.0 mmol/L)	Rosuvastatin 20 mgvs placebo	486	8901	8901	270	216
SPARCL	3803/4731	4·9	Two fasting glucose values≥7.0 mmol/L and at leastpost-baseline glucose≥2 mmol/l above baseline	Atorvastatin 80 mgvs placebo	281	1905	1898	166	115
HPS	14573/20536	5.0	Physician reported; medication	Simvastatin 40 mgvs placebo	628	7291	7282	335	293
ASCOT-LLA	7773/10305	3.3† ‡	WHO 1999 criteria	Atorvastatin 10 mgvs placebo	288	3910	3863	154	134
CORONA	3534/5011	2.7† ‡	Physician reported	Rosuvastatin 20 mgvs placebo	188	1771	1763	100	88
PROSPER	5181/5804	3.2	One fasting glucose value>7.0 mmol/L; medication	Pravastatin 40 mgvs placebo	292	2588	2593	165	127
GISSI-HF	3378/4574	3.9‡	Two fasting glucose values≥7.0 mmol/L	Rosuvastatin 10 mgvs placebo	440	1660	1718	225	215
4S	4243/4444	5.4‡	Physician reported; medication;one fasting glucose value≥7.0 mmol/L	Simvastatin 20–40 mgvs placeb	391	2116	2127	198	193
WOSCOPS	5974/6595	4.8	Two fasting glucose values≥7.0 mmol/L; medication	Pravastatin 40 mgvs placebo	168	2999	2975	75	93
LIPID§	6997/9014	6.0	One fasting glucose value≥7.0 mmol/L; medication	Pravastatin 40 mgvs placebo	264	3496	3501	126	138
AFCAPSTexCAPS	6211/6605	5.2†	Physician reported;medication;one fasting glucosevalue ≥7.0 mmol/L	Lovastatin 20–40 mgvs placebo	146	3094	3117	72	74
ALLHAT-LLT	6087/10355	4.8†	One fasting glucose value≥7.0 mmol/L	Pravastatin 40 mgvs no treatment	450	3017	3070	238	212
MEGA	6086/7832	5.3	Physician reported; medication;two fasting glucosevalues ≥7.0 mmol/L	Pravastatin 10–20 mgvs no treatment	33	3013	3073	172	164
GISSIPREVENZIONE	3460/4271	2.0‡	One fasting glucosevalue ≥7.0 mmol/L	Pravastatin 20 mgvs no treatment	201	1743	1717	96	105

DM = diabetes mellitus; OGTT = oral glucose tolerance test; WHO = World Health Organization;

† Data from total cohort (including diabetes at baseline).

‡ Median.

§ Includes only patients with normal fasting glycaemia at baseline.

**Table 2:** Characters for non-diabetic participants in 14 trials that reported incident diabetes.

	MeanAge (yr)	MeanBMI(kg/m^2^)	Sex(men %)	PriorCHD (%)	PriorStroke (%)	Hypertension(%)	SystolicBP/DiastolicBP (mm Hg)	Smoking(current %)
JUPITER	66.0‡	28.4‡	61.8	0.0	Not reported	0.0	134.0/80.0	15.8
SPARCL	62.7	27.4	59.7	0.0	69.1	61.9	138.6/81.7	19.2
HPS	64.7	27.2	77.6	78.3	Not reported	41.6	143.0/81.0	14.7
ASCOT-LLA	63.1†	28.6†	81.1	0.0	9.7	100.0	164.2/95.0	32.7
CORONA	73.0†	27.0†	76.5	100.0	12.5	63.4	129.0/76.0	8.6
PROSPER	75.3	26.8	48.3	44.0	11.2	61.9	154.6/83.8	26.8
GISSI-HF	68.0	27.1	77.4	100.0	4.5	54.3	127.0/77.0	14.1
4S	58.6	26.0	81.4	100.0	Not reported	26.0	138.8/83.5	25.6
WOSCOPS	55.2	26.0	100.0	0.0	Not reported	15.7	135.5/84.0	44.0
LIPID§	62.0‡	NA	83.2	100.0	4.1	41.7	Not reported	9.6
AFCAPS TexCAPS	58.0†	27.0†	85.0	0.0	Not reported	22.0	138.0/78.0	12.4
ALLHAT-LLT	66.3	29.9	51.2	14.2	Not reported	100.0	145.0/84.0	23.2
MEGA	58.3	23.8	31.6	0.0	0.0	41.8	132.2/78.6	20.6
GISSIPREVENZIONE	59.3	26·5	86.3	100.0	Not reported	37.0	Not reported	11.8

BMI = body mass index; CHD = coronary artery heart disease; BP = blood pressure;

† Data from total cohort (including diabetes at baseline).

‡ Median.

§ Includes only patients with normal fasting glycaemia at baseline.

**Table 3:** Characters for non-diabetic participants in 14 trials that reported incident diabetes.

	BaselineLDL-c(mmol/l)	BaselineHDL-c(mmol/l)	BaselineTotalCholesterol(mmol/l)	BaselineTriglycerides(mmol/l)	Relative (%)LDL-CReduction*	Relative (%)HDL-cReduction*	Relative (%)Total CholesterolReduction*
JUPITER	2.8	1.3	4.8	3.0	50.0 (12 months)	0.0	Not reported
SPARCL	3.4	1.3	5.4	3.7	42.1 (average in trial)	2.3	28.8
HPS	3.4	1.1	5.9	2.0	29.4 (average in trial)	Not reported	Not reported
ASCOT-LLA	3.4	1.3	5.5	1.7	34.8 (12 months) †	−1.5†	18.2†
CORONA	3.5	1.2	5.4	2.0	45.1 (3months)†	5.0†	Not reported
PROSPER	3.8	1.3	5.7	1.5	30.7 (12 months)	5.0	Not reported
GISSI-HF	3.2	Not reported	Not reported	Not reported	34.9 (12 months)	Not reported	Not reported
4S	4.9	1.2	6.7	1.5	36.7 (12 months)	7.0	26.0
WOSCOPS	4.9	1.1	7.0	4.2	23.7 (12 months)	5.0	20.0
LIPID§	3.8	0.9	5.6	3.9	25 (during 5 years)	5.0	17.8
AFCAPS TexCAPS	3.9	1.0	5.7	1.8	26.7 (12 months)	6.0	18.0
ALLHAT-LLT	3.7	1.2	5.7	3.9	18.1 (24 months)	7.1	8.4
MEGA	4.1	1.5	6.3	1.4	17.1 (12 months)	5.0	9.0
GISSIPREVENZIONE	3.9	1.2	5.9	4.3	11.5 (12 months)	0.6	7.9

LDL-c = low-density lipoprotein cholesterol; HDL-c = high-density lipoprotein cholesterol;

*Difference between the groups in the change from baseline to timepoint in LDL-C.

† Data from total cohort (including diabetes at baseline).

§ Includes only patients with normal fasting glycaemia at baseline.

**Table 4:** Characters for non-diabetic participants in 14 trials that reported incident diabetes.

	Relative (%)TriglyceridesReduction*	EndpointLDL-C(mmol/l)(statin group)	Aspirin(%)	ACEinhibitor (%)	Beta-blocker(%)	Cardiovascularevents(statin vs placebo)	Total CHDcoronary events(statin vs placebo)	Jadadscore
JUPITER	16.1	1.4	16.6	Not reported	Not reported	142/251	Not reported	6
SPARCL	10.6	1.6	87.3	28.5	17.7	864/1094	305/475	4
HPS	Not reported	2.4	Not reported	Not reported	Not reported	Not reported	619/835	4
ASCOT-LLA	12.6†	2.2	17.0	Not reported	Not reported	389/486	178/247	6
CORONA	20.5†	2.0	Not reported	80.0	75.2	1104/1164	554/588	5
PROSPER	13.0	2.5(3 months)	Not reported	Not reported	Not reported	454/523	Not reported	7
GISSI-HF	Not reported	2.2	45.1	77.6	62.4	1305/1283	Not reported	7
4S	17.0	3.0	36.8	Not reported	56.8	136/207(death)	353/502	4
WOSCOPS	12.0	3.6	Not reported	Not reported	Not reported	50/73(death)	174/248	4
LIPID§	11.0	2.9	82.3	16.0	47.1	331/433(death)	287/373(death)	4
AFCAPS TexCAPS	15.0	2.9	17.1	7.6	4.5	194/255	163/215	4
ALLHAT-LLT	3.0	2.7	30.9	Not reported	Not reported	295/300(death)	380/421	2
MEGA	13.0	3.2	1.0	12.6	8.3	125/172	66/101	4
GISSIPREVENZIONE	4.7	3.2	78.7	41.5	42.9	101/113(death)	31/49(death)	3

LDL-c = low-density lipoprotein cholesterol; CHD = coronary artery heart disease;

*Difference between the groups in the change from baseline to timepoint in LDL-C.

† Data from total cohort (including diabetes at baseline).

§ Includes only patients with normal fasting glycaemia at baseline.

### Statistical analysis

The 14 trials were stratified according to the target LDL-c level of <1.8 mmol/L, >2.59 mmol/L, and 1.8–2.59 mmol/L. Odds ratio (OR) and 95% confidence interval (CI) were used to compare the mean differences in each subgroup separately. *I* ^2^ statistic, which is derived from Cochran’s *Q* [100×(*Q*–*df*/*Q*)] and provides a measure of the proportion of overall variation attributable to between-trial heterogeneity, was used to quantify statistical heterogeneity between trials [27]. Random-effects models were selected for a more conservative assessment (i.e., wide CIs) of the average effect size. Moreover, an independent analysis restricted to trials of standard LDL-c lowering with statin therapy was carried out (trials did not meet either of the following requirement: (1) target LDL-c level ≤2.59 mmol/L or (2) relative LDL-c reduction of at least 30% of baseline). Meta-regression analyses were used to identify the risk factors of incident diabetes between trials. Stata version 11.0 was used to analyze the data, and *P*<0.05 was considered statistically significant. Sensitivity analyses were also carried out. A funnel plot and Egger test were used to estimate publication bias [28].

## Results

### New-onset diabetes stratified with target levels of LDL-c

**Figure 1:**
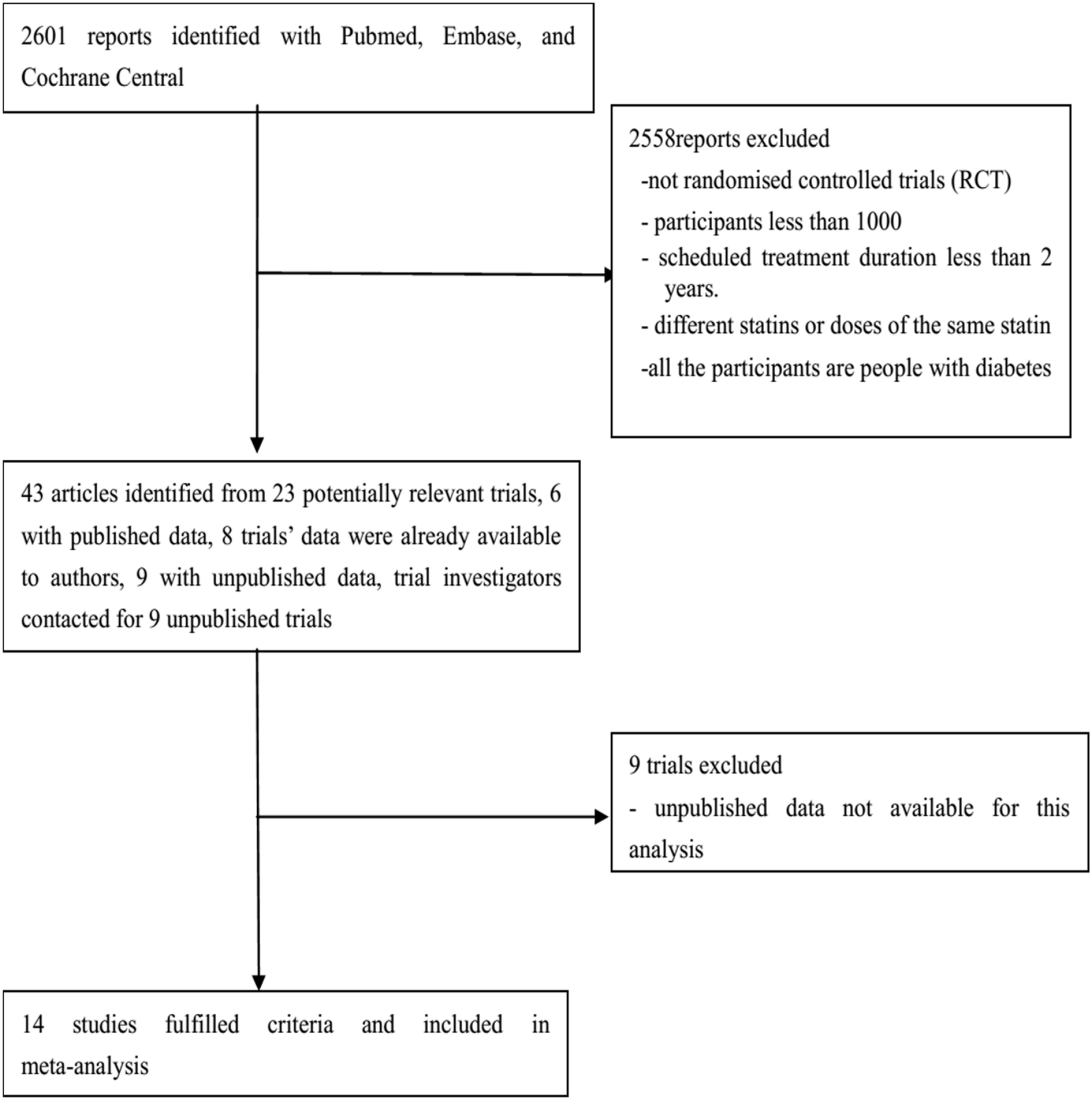
Flow diagram of literature search to identify randomised placebo-controlled and standard care-controlled statin trials.

**Figure 2:**
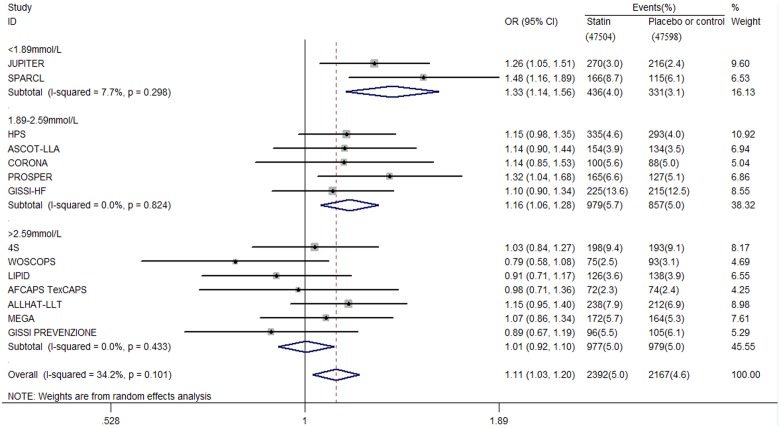
Association between different target LDL-c level and incident diabetes.

Fourteen eligible trials with a total of 95102 non-diabetic participants were included (Figure 1). The characteristics of the trials are shown in Table 1 to Table 4. Study quality was generally high, 12 (86%) of 14 trials had a Jadad score of ≥4. A noticeable effect of statin therapy on new-onset diabetes was observed when their intensified target LDL-c level was lower than 1.8 mmol/L (OR 1.33, 95% CI 1.14 to 1.56; *I* ^2^ = 7.7%) and within 1.8 mmol/L to 2.59 mmol/L (OR 1.16, 95% CI 1.06 to 1.28; *I* ^2^ = 0.0%) (Figure 2). However, the risk of incident diabetes did not increase when the target LDL-c level was higher than 2.59 mmol/L (OR 1.01, 95% CI 0.92 to 1.10; *I* ^2^ = 0.0%) (Figure 2). In absolute terms, one additional case of diabetes is diagnosed per 103 patients and per 141 patients whose target LDL-c was ≤1.8 mmol/L and within 1.8 mmol/L to 2.59 mmol/L when taking statin therapy for 4 years.

### New-onset diabetes in standard LDL-c lowering group

**Figure 3:**
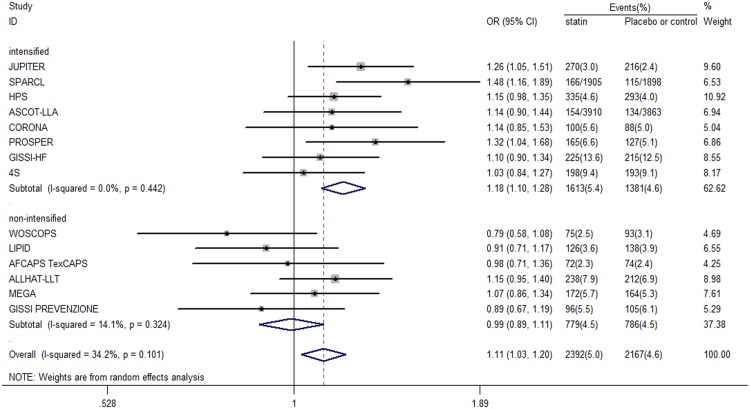
Association between intensified and non-intensified LDL-c level and incident diabetes.

Among the six trials in the standard LDL-c lowering group, no individual trial showed a positive effect of statin therapy on incident diabetes. In the combined data set, no clear association was found between standard LDL-c lowering with statin therapy and incident diabetes compared with placebo or standard care control therapy (OR 0.99, 95% CI 0.89 to 1.11) (Figure 3). The heterogeneity between trials was low (χ^2^ for heterogeneity = 5.82; *P* = 0.324; *I* ^2^ = 14.1%), which indicates that most variations were attributable to chance alone.

### Meta-regression analyses

**Figure 4:**
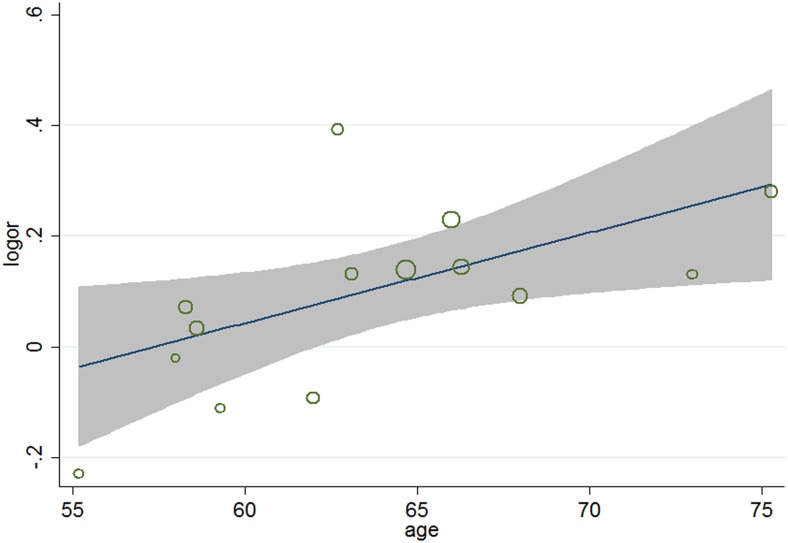
Meta-regression of baseline age for incident diabetes.

**Figure 5:**
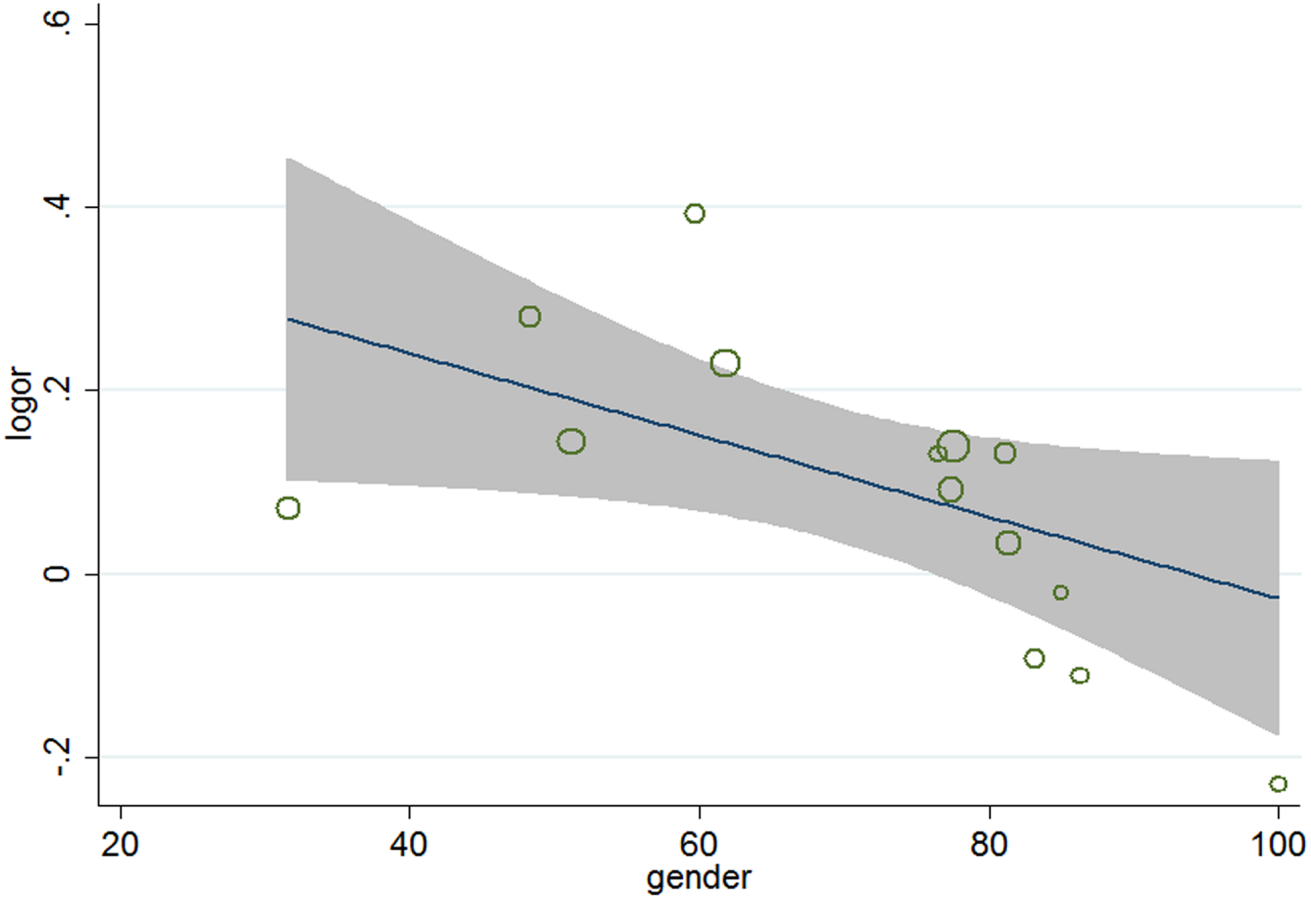
Meta-regression of gender for incident diabetes.

**Figure 6:**
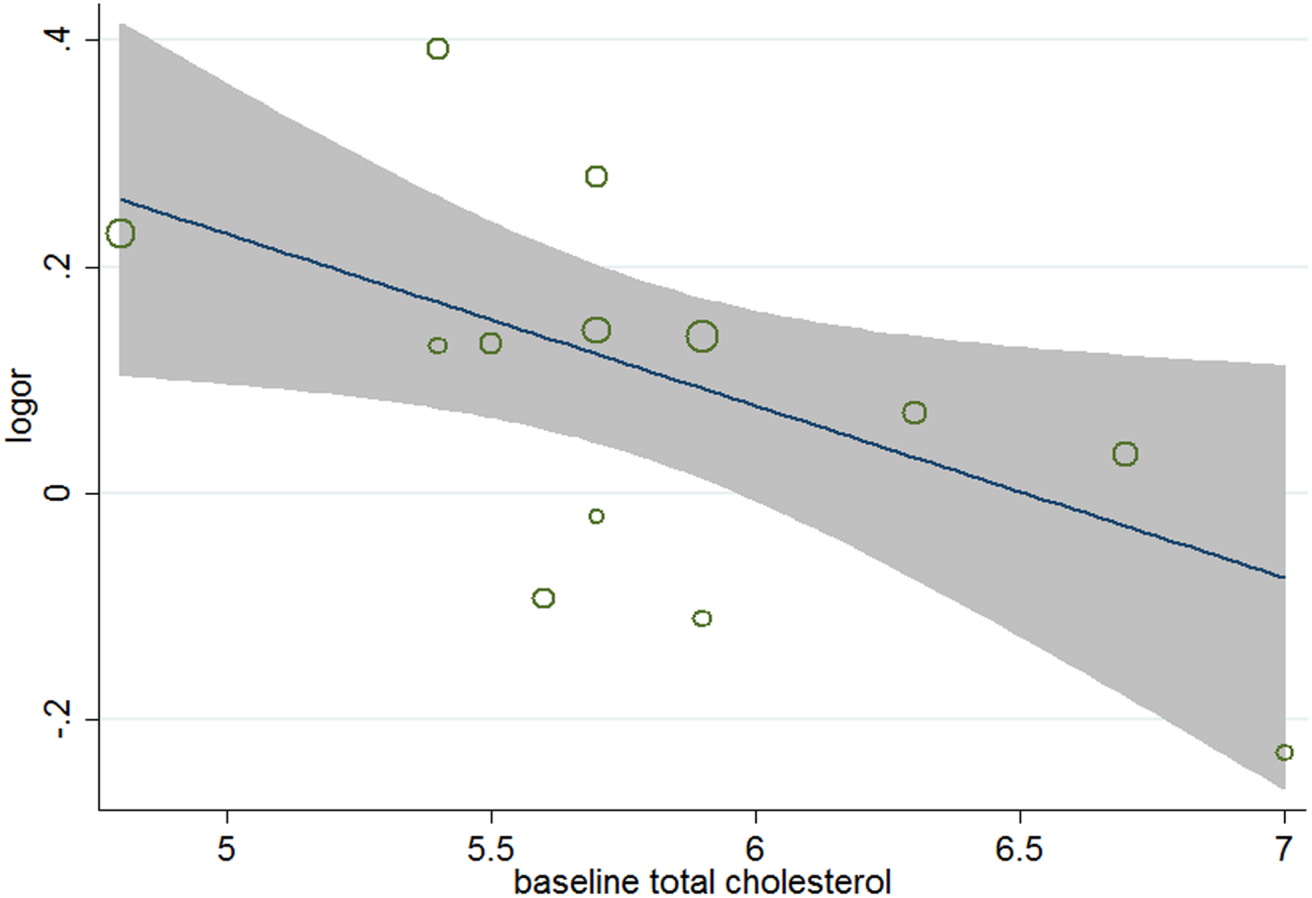
Meta-regression of baseline cholesterol for incident diabetes.

**Figure 7:**
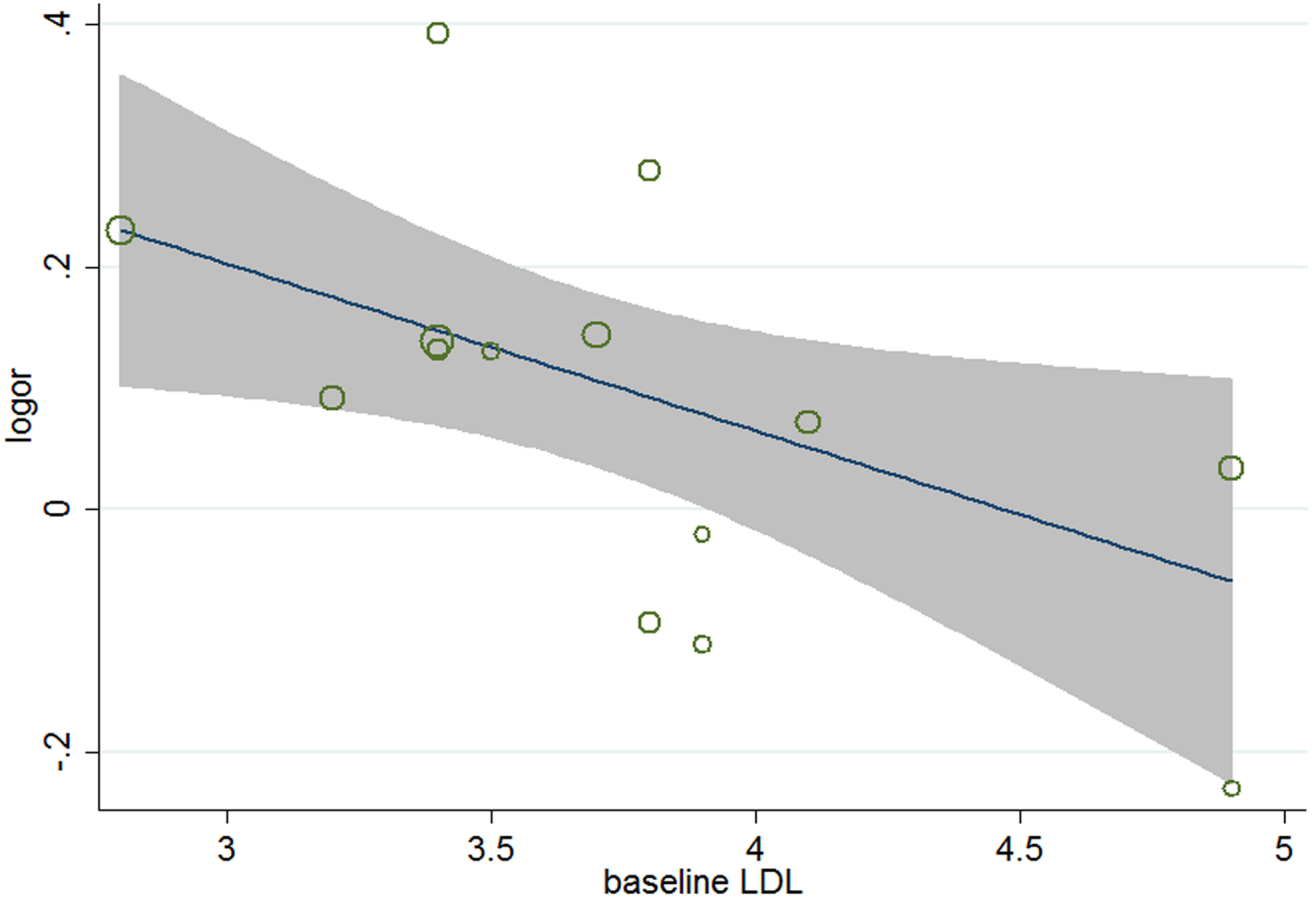
Meta-regression of baseline LDL for incident diabetes.

**Figure 8:**
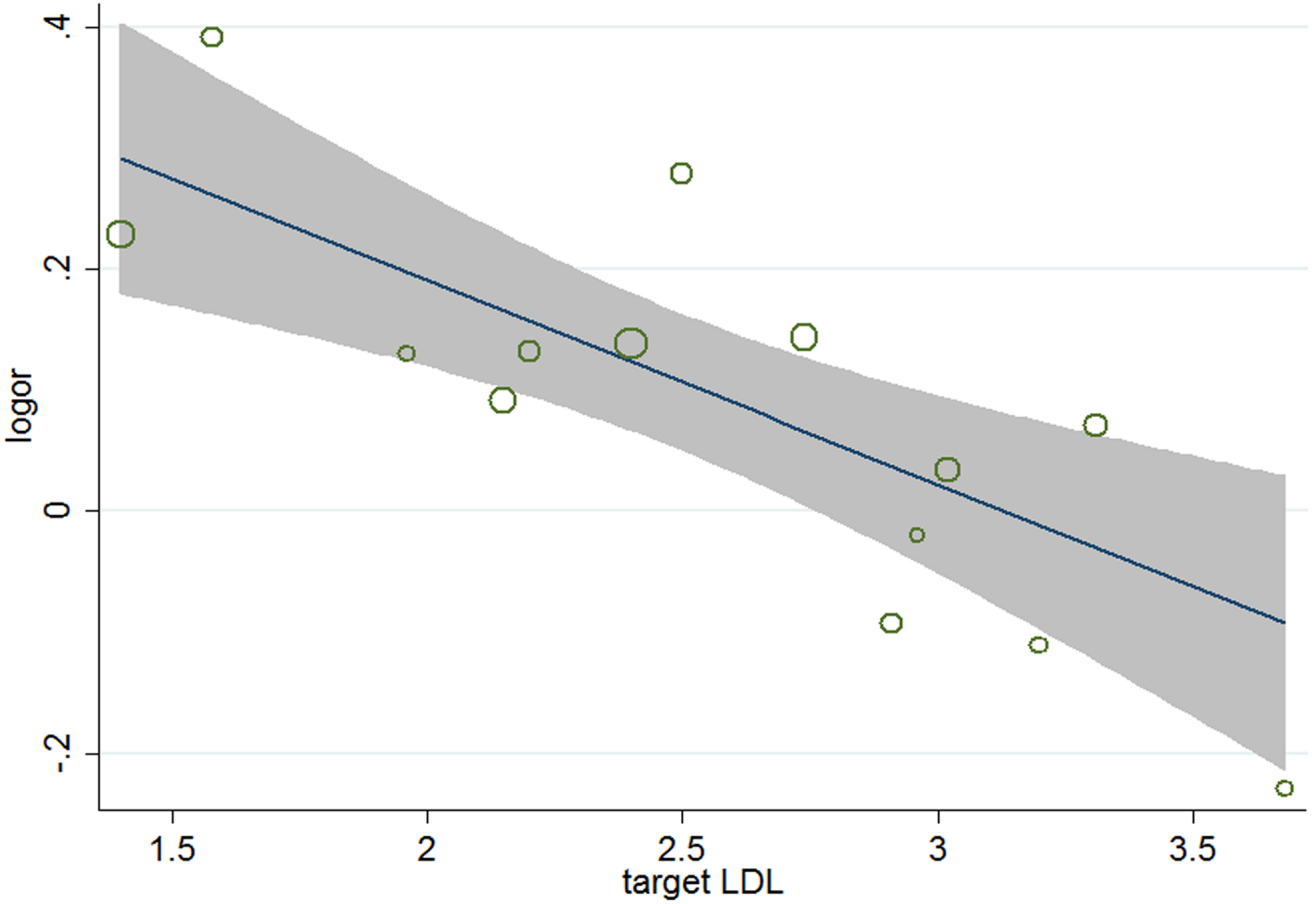
Meta-regression of target LDL for incident diabetes.

**Figure 9:**
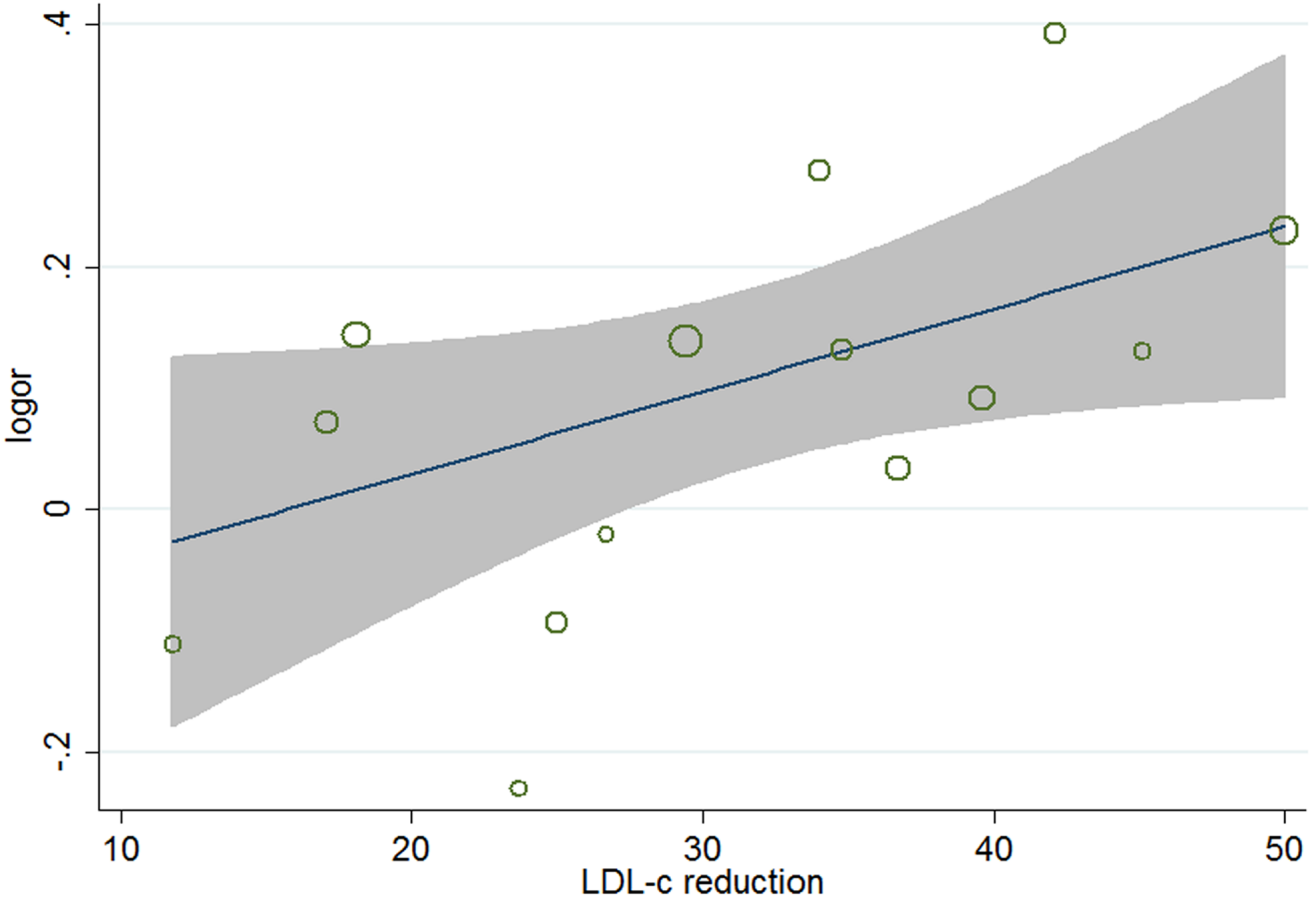
Meta-regression of LDL reduction for incident diabetes.

To identify other potential factors of the residual difference between the 14 trials (*χ* ^2^ for heterogeneity = 19.77; *P* = 0.101; *I* ^2^ = 34.2%), meta-regression analyses were carried out. The results (Table 5) showed that aside from age (*P* = 0.026, 95% CI 1.002 to 1.031) (Figure 4), gender (*P* = 0.038, 95%, CI 0.991 to 0.999) (Figure 5), and baseline total cholesterol (*P* = 0.034, 95% CI 0.748 to 0.967) (Figure 6), baseline level of LDL-c (*P* = 0.031, 95% CI, 0.771 to 0.985) (Figure 7), target LDL-c level (*P* = 0.005, 95% CI, 0.758 to 0.941) (Figure 8), and relative LDL reduction (*P* = 0.044, 95% CI, 1.021 to 3.907) (Figure 9) were risk factors of new-onset diabetes after statin therapy.

**Table 5:** Meta-regression of baseline characters for incident diabetes.

logor	t	P>|t|	[95% Conf. Interval]
**age**	2.54	0.026	1.002305 1.030816
**sex**	−2.34	0.038	.9912401.9996951
**target LDL**	−3.40	0.005	.7582104.941228
**baseline LDL**	−2.45	0.031	.771076.9849836
**baseline cholesterol**	−2.41	0.034	.7478781.9867268
**LDL reduction**	2.25	0.044	1.000208 1.013721
**dose**	1.25	0.235	.9979259 1.007709
**BMI**	1.29	0.224	.9773928 1.091429
**CHD**	−1.21	0.250	.9971045 1.000831
**stroke**	2.37	0.064	.9995688 1.010495
**hypertension**	0.98	0.346	.9983291 1.004423
**systolic BP**	0.69	0.506	.9939916 1.011491
**diastolic BP**	0.05	0.962	.9809797 1.020278
**smoking**	−0.34	0.742	.987824 1.009013
**baseline HDL**	1.87	0.088	.9145669 3.037582
**baseline triglycerides**	−0.85	0.414	.8823282 1.057124
**Aspirin**	−0.23	0.823	.9957346 1.003503
**Aceinhibitor**	0.38	0.718	.9945277 1.007432
**Beta-block**	−0.46	0.663	.9929148 1.004881
**HDL Increase**	−0.75	0.470	.9485923 1.026537
**cholesterol reduction**	0.82	0.437	.987558 1.02625
**triglycerides reduction**	0.36	0.726	.9799687 1.028448

### Sensitivity analyses and publication bias

Sensitivity analyses showed that the results from fixed-effects model (OR 1.33, 95% CI 1.15 to 1.54, *I* ^2^ = 7.7%) were similar to random-effects model meta-analysis for incident diabetes when the target LDL-c was ≤1.8 mmol/L. Fixed-effects model meta-analysis (OR 1.16, 95% CI 1.06 to 1.28, *I* ^2^ = 0%) and result, except for PROSPER trial for its positive effect (OR 1.13, 95% CI 1.02 to 1.26, *I* ^2^ = 0%), produced similar results to original analysis for new-onset diabetes when the target LDL-c was within 1.8 mmol/L to 2.59 mmol/L. A funnel plot and Egger test (*P* = 0.683, 95% CI −4.140 to 5.504) indicated that the publication bias of this analysis from the five trials was weak. For analysis that targets LDL-c higher than 2.59 mmol/L, the result restricted to placebo-controlled trials (OR 0.95, 95% CI 0.83 to 1.08, *I* ^2^ = 0%), and the result without ALLHAT-LLT for its biggest weight (OR 0.97, 0.87 to 1.07, *I* ^2^ = 0%) was similar to the primary analysis. The result of the fixed-effects model analysis was also consistent with the random-effects model meta-analysis when pooling data from the six trials. A funnel plot and Egger test (*P* =  0.046, 95% CI −8.415 to −0.120) revealed that an apparent asymmetry suggested the presence of a potential publication bias, a language bias, inflated estimates by a flawed methodologic design in smaller studies, and/or a lack of publication of small trials with opposite results.

## Discussion

Aggressive LDL-c lowering based on coronary heart disease (CHD) risk reflected the potential extra benefits. Lowering LDL-c to less than 2.6 mmol/L and further lower level, e.g., 1.8 mmol/L, was recommended for those with known CHD or at very high risk. In addition, a minimum LDL-c reduction of 30% to 40% was suggested for those considered to be at moderate to very high risk for CHD [29]. A goal target is not always achievable through increasing the dose of statin drug. Doubling the statin dose is associated with an approximately 5% to 6% greater lowering of LDL-c [7], and toxicity is often dose related. A published meta-analysis reported that an intensive dose statin therapy was associated with a higher incidence of T2DM [5]. Given the potential effect of statin drug on new-onset T2DM, we were interested in the relationship between target LDL-c level after statin treatment and new-onset T2DM. The included studies were stratified by the target LDL-c level. The main findings were that statin drugs with lower intensified target LDL-c level led to higher risk of incident diabetes. Incident diabetes did not increase when the target LDL-c level was higher than 2.59 mmol/L. Aside from age, female, and base level of total cholesterol, meta-regression analysis showed that target and baseline levels of LDL-c and relative LDL-c reduction were predictors of statin-induced diabetes.

The obtained results seem at variance with a previous study [5] in that target LDL-c level and relative LDL-c reduction, but not the dose of statin drug, accounted for risk factors of statin-induced diabetes. The level of LDL-c significantly decreased by more than 30% compared with the baseline among most of the intensive dose statin studies in the previous meta-analysis. However, the participants were only stratified by the dose rather than the target goal statistically. Thus, recognizing whether statin-induced diabetes is related with LDL-c level or not from the previous study is difficult. The comparison of the three studies [intensive vs moderate dose statin study [5], intensive LDL-c lowering with statin (1.8≤LDL-c ≤2.59 mmol/L) vs placebo study, intensive LDL-c lowering with statin (LDL-c≤1.8 mmol/L) vs placebo study] showed that the relative LDL-c reductions were 12% to 22%, 29.4% to 45%, and 42% to 50%. The corresponding increased risks of statin-induced diabetes were 12%, 16%, and 33%. According to this stratified LDL-c target goal analysis, doses of statins in trials with intensified target LDL-c levels ≤1.8 mmol/L and within 1.8 mmol/L to 2.59 mmol/L were almost similar (10 mg/d to 40 mg/d), but the risk of diabetes was elevated by 17% as the LDL-c concentration decreased to approximately 0.79 mmol/L. For trials of aggressive LDL-c lowering, the dose of statin drugs was also approximately similar to the standard LDL-c lowering trials ranging from 10 mg to 40 mg per day. However, the relative LDL-c reduction was apparently higher, and the target LDL-c level was lower than the standard LDL-c lowering trials. For the intensive LDL-c lowering trials, LDL-c reduction ranged from 29.4% to 50%, and the target LDL-c level ranged from 1.4 mmol/L to 3.0 mmol/L. For the standard LDL-c lowering trials, the LDL-c reduction ranged from 11.8% to 26.7%, and the target LDL-c level ranged from 2.7 mmol/L to 3.6 mmol/L. Therefore, the exact opposite results revealed that LDL-c reduction may be a relevant factor of statin-induced diabetes. Our findings are inconsistent with the meta-regression analyses of Naveed [4] and Eliano Pio Navarese [30]. The risk for developing diabetes was affected by the ability of statins to reduce cholesterol. The differences between our study and that of Naveed et al. are accounted in the supplementary trial (SPARCL). The SPARCL trial [13] reached a relatively higher reduction of baseline LDL-c and T2DM incidence than other trials in the statin group. The replenishment of significant positive result possibly changed the whole findings. The latter meta-regression analysis recruited the trials and administered one kind of statin with unvaried dose, which may lead to aggregation bias but a relatively better homogeneity.

Less progress has been made in elucidating the mechanisms of statin-induced diabetes. Although still experimental, the potential mechanisms of statin-induced T2DM are associated with LDL-c concentration and the abilities of statins to reduce LDL-c, which are obtained from our study. A possible suggestion is that patients are aware of their treatment allocation during follow up. Those patients with substantially reduced LDL-c will be complacent and assume poorer lifestyles, gain weight, and then develop diabetes [31]. Potential plausible molecular explanations for statin-induced diabetes include impairment in insulin secretion and exacerbation of insulin resistance [32]. HMG CoA reductase inhibitors competitively inhibit the activity of HMG CoA reductase and result in a transient, modest decrease in cellular cholesterol concentration [33]. The decrease in cholesterol concentration activates a cellular signaling cascade culminating in the activation of sterol regulatory element binding protein. This protein is a transcription factor that upregulates the expression of the gene encoding the LDL receptor, which increase uptake of circulating LDL-c [34]. Thus, the more reduction of plasma LDL-c indicates higher HMG CoA reductase inhibited. The more inhibition of HMG-CoA reductase suppresses the synthesis of isoprenoids, which can significantly upregulate insulin-responsive glucose transporter-4, which leads to serious impaired glucose uptake [35]. The high glucokinase inhibition by abundance of plasma-derived LDL-c is another potential biochemical explanation for the observed increase of new-onset diabetes with lower plasma LDL-c [36]. Glucokinase is associated with the cascade of closure of ATP-dependent potassium channel, depolarization, and calcium influx that leads to insulin secretion [37], [38]. Similar to isoprenoids, ubiquinone (CoQ10) synthesis also decreases further as the reduction of circulating LDL-c increases. CoQ10 is an essential factor in the mitochondrial electron-transfer system. Thus, the decrease of CoQ10 will result in inhibition of insulin secretion because of reduced ATP production [35], [39]. In addition, the superabundant oxidation of LDL-c inside the cell may incite an inflammatory cascade. The interplay among inflammation, oxidation, and apoptosis within the β-cells, which is potentially triggered by increased abundance of cell membrane-derived LDL-c caused by the statin-induced inhibition of de-novo cholesterol synthesis [4], [35], [40], could explain higher risk of incident diabetes with lower LDL-c level.

Some limitations exist in this analysis. First, only two trials reached the target LDL-c level less than 1.8 mmol/L; thus, the effect size derived from the two trials was not sufficient and with less comparability. Second, the diagnosis of incident diabetes varied among trials like other similar studies. Third, our analysis had missing data from other large-scale trials, which lowered the statistical power. Notably, the original authors were contacted for unpublished information, but no response was received.

## Conclusions

Aside from age, female, and baseline level of total cholesterol, meta-regression analyses showed that target and baseline levels of LDL-c and relative LDL-c reduction were predictors of statin-induced diabetes. The lower intensified target LDL-c level of statin therapy contributed to the higher risk of diabetes. Although the cardiovascular benefit from cholesterol-lowering statin drugs overweighs the diabetes risk, incident diabetes should be considered to weigh the pros and cons when LDL-c reaches a lower level, e.g., less than 1.8 mmol/L, especially in primary prevention low-risk patients.

## Supporting Information

Checklist S1 PRISMA Checklist.
(DOC)
